# HIV and hepatitis C co-infection in Europe, Israel and Argentina: a EuroSIDA perspective

**DOI:** 10.1186/1471-2334-14-S6-S13

**Published:** 2014-09-19

**Authors:** Lars Peters, Amanda Mocroft, Jens Lundgren, Daniel Grint, Ole Kirk, Jürgen Kurt Rockstroh

**Affiliations:** 1 CHIP, Department of Infectious Diseases and Rheumatology, Rigshospitalet, University of Copenhagen, Denmark; 2 University College Medical School, London, UK; 3 University of Bonn, Department of Medicine I, Bonn, Germany

## Introduction

The EuroSIDA observational cohort study was initiated in 1993 by Jens Lundgren (Copenhagen HIV Programme) and Andrew Phillips (University College London) as the successor of the AIDS in Europe Study, with the main objective of assessing the impact of antiretroviral drugs on outcomes for the general population of HIV-infected patients living in Europe. As of April 2014 EuroSIDA included 18,786 patients followed at 111 hospitals in 34 European countries plus Argentina and Israel. Since its inception, EuroSIDA has provided data published in 202 papers in peer-reviewed journals, among them 14 papers on hepatitis C virus (HCV) co-infection [1-14] .

As the HIV epidemic has evolved and the incidence of AIDS has decreased, the focus of EuroSIDA has shifted to addressing the research questions most pertinent to the clinical care of Europeans living with HIV on a long-term basis. With more than a quarter of all EuroSIDA patients positive for antibodies to HCV, and with growing concern about the burden of liver-related disease and death, hepatitis C research has come to play an increasingly important role in EuroSIDA in recent years.

This article will describe key characteristics of the HIV/HCV co-infected population in EuroSIDA, and summarize the most important EuroSIDA co-infection studies.

## Study design of EuroSIDA

**Figure 1:**
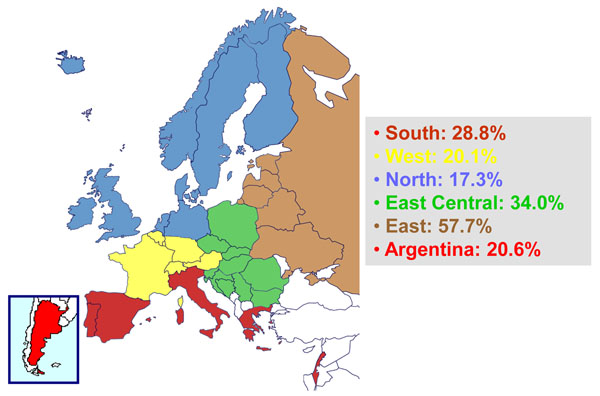
Anti-HCV antibody prevalence in different EuroSIDA regions

Patients eligible for enrolment in EuroSIDA are HIV-1 infected individuals aged 16 and older. To ensure that included patients are representative for patients followed at the participating clinics, EuroSIDA has, enrolled a new cohort of around 1200-2500 randomly selected patients every second year. EuroSIDA is the only cohort of HIV-infected individuals that include patients from all European geographical regions including the Russian Federation and other countries of the former Soviet Union (Figure 1). Comparisons of regional differences in clinical outcomes are therefore important to most EuroSIDA studies.

Extensive demographic and clinical data are collected at the date of enrolment and prospectively at six-month intervals from all EuroSIDA patients at clinical sites. These data are sent to the coordinating center at CHIP, a WHO Collaborating Centre based at the Department of Infectious Diseases and Rheumatology at Rigshospitalet, in Copenhagen, Denmark. Complete information about the data items collected is available at http://www.cphiv.dk.

Data have been collected on HCV antibody and HCV-RNA status since 1997 and on HCV genotype since 2005. Routine liver biochemistry has been collected since 1999. Information on hepatic encephalopathy, hepatocellular carcinoma and other manifestations of decompensated liver disease has been collected since 1998, 2001 and 2010, respectively. Data on liver biopsy and Fibroscan® have been collected since 2010, with sites requested to list all previous tests and return the histological report for internal validation. The METAVIR classification system is used for determining the liver fibrosis stage.

One of the major strengths of EuroSIDA is the establishment of an extensive quality assurance system that includes data checking at the coordinating office, as well as regular monitoring visits with source verification of all new major clinical events, plus a random selection of individuals followed at the clinics. Detailed information on the cause of death are collected using the “Coding of Death in HIV” (CoDe) system [15].

The central plasma repository, which was set up in 1997, receives plasma from most EuroSIDA patients at six-month intervals. In 2006, all patients with unknown hepatitis B and C status and stored plasma samples were identified and tested in a central reference laboratory for anti-HCV antibodies, HCV-RNA and genotype, as well as for relevant hepatitis B and D markers. This has established a cohort of almost 2,000 HCV antibody-positive patients who are well characterized with regards to HCV-RNA and genotype. The plasma repository has also been used to test all HCV antibody positive and hepatitis B surface antigen positive patients for plasma hyaluronic acid, a marker of liver fibrosis.

## Epidemiology of HIV/HCV co-infection in EuroSIDA

Among the 18,786 HIV-positive individuals enrolled in EuroSIDA, 16,371 have been tested for HCV antibodies and 5,298 patients (32.4% (95% CI 31.6 - 33.1)) are known to be positive. Of those, 3,645 (68.8%) have been tested for HCV-RNA and 2,571 (70.5% (95% CI 69.1 – 72.0)) were positive for it.

In 2008, the epidemiological and virological characteristics of 1,940 HCV antibody-positive EuroSIDA patients were published [14]. These patients had been tested centrally for HCV-RNA and genotype. The cohort included 1,496 patients (77%) who tested positive for HCV-RNA. Among these patients, 786 people (53%) were infected with genotype 1, while 4%, 29% and 15% were infected with genotypes 2, 3 and 4, respectively. Patients who had cleared HCV-RNA spontaneously were more likely to be female and infected with hepatitis B virus, whereas people who inject drugs (PWID) were less likely to have cleared HCV-RNA.

Table 1 describes the characteristics of the 3,030 HCV antibody-positive patients currently under follow-up in the clinics who continue to contribute data to EuroSIDA. The prevalence of HCV antibody-positivity varies across different geographic regions. In Eastern and Southern Europe, where HIV is often acquired through injecting drug use (IDU), 58% and 29% of patients are also HCV antibody positive, respectively. In Northern and Western Europe, where sexual transmission among men who have sex with men (MSM) is the predominant mode of HIV transmission, 17% and 20% of patients are HCV antibody positive, respectively (figure 1). 61% of all HCV antibody positives have reported IDU as the most likely mode of HIV transmission, while for 19% and 13% the main risk factor is heterosexual and homosexual contact, respectively. The median age is 44 years and two thirds are men. Nearly 85% are receiving combination antiretroviral therapy (cART) and among them 90.1% have an HIV-RNA below 500 copies/mL. Around a quarter of all patients have received interferon based therapy.

**Table 1:** Characteristics of HCV antibody positive patients currently under follow-up in EuroSIDA

		***HCV RNA Status***			
		
	***Total (N=3030)***	***Negative (N=837)***	***Positive (N=1370)***	***Unknown (N=823)***	P-value*
***Sex***					
Female	992 (32.8%)	233 (23.5%)	443 (44.7%)	316 (31.9%)	0.1870
Male	2031 (67.2%)	524 (25.8%)	919 (45.2%)	588 (29.0%)	
***Age***					
Median (IQR)	44 (36 - 50)	47 (39 - 52)	47 (38 - 51)	39 (34 - 46)	<.0001
***CD4 Cell Count***					
Median (IQR)	512 (340 - 712)	572 (413 - 753)	525 (347 - 723)	445 (273 - 640)	<.0001
***Transmission Risk***					
MSM	387 (12.8%)	147 (38.0%)	151 (39.0%)	89 (23.0%)	<.0001
IDU	1836 (60.7%)	417 (22.7%)	909 (49.5%)	510 (27.8%)	
Heterosexual	574 (19.0%)	123 (21.4%)	200 (34.8%)	251 (43.7%)	
Other	226 (7.5%)	70 (31.0%)	102 (45.1%)	54 (23.9%)	
***Race***					
Non-White	259 (8.6%)	94 (36.3%)	123 (47.5%)	42 (16.2%)	<.0001
White	2764 (91.4%)	663 (24.0%)	1239 (44.8%)	862 (31.2%)	
***Region of EuroSIDA***					
South	719 (23.8%)	207 (28.8%)	391 (54.4%)	121 (16.8%)	<.0001
West C	437 (14.5%)	162 (37.1%)	224 (51.3%)	51 (11.7%)	
North	353 (11.7%)	129 (36.5%)	186 (52.7%)	38 (10.8%)	
East C	502 (16.6%)	137 (27.3%)	248 (49.4%)	117 (23.3%)	
East	926 (30.6%)	108 (11.7%)	282 (30.5%)	536 (57.9%)	
Argentina	86 (2.8%)	14 (16.3%)	31 (36.0%)	41 (47.7%)	
***HBsAg Status***					
Negative	2719 (89.9%)	681 (25.0%)	1212 (44.6%)	826 (30.4%)	0.0528
Positive	172 (5.7%)	52 (30.2%)	78 (45.3%)	42 (24.4%)	
Unknown	132 (4.4%)	24 (18.2%)	72 (54.5%)	36 (27.3%)	
***Taking cART***					
No	467 (15.4%)	92 (19.7%)	191 (40.9%)	184 (39.4%)	<.0001
Yes	2556 (84.6%)	665 (26.0%)	1171 (45.8%)	720 (28.2%)	
***HIV RNA Viral Load(Among those taking cART)***					
<500copies/ml	2302 (90.1%)	645 (28.0%)	1079 (46.9%)	578 (25.1%)	<.0001
***Interferon Treatment***					
Previously Exposed	800 (26.5%)	367 (45.9%)	397 (49.6%)	36 (4.5%)	<.0001

*P-value from Kruskal-Wallis or Chi-square test

IDU: injecting drug use; MSM: men who have sex with men; HBsAg: hepatitis B surface antigen, cART: combination antiretroviral therapy

## Acute hepatitis C and risk of HCV reinfection

Since HCV is more transmissible than HIV through blood contact, most PWID are already HCV-infected by the time they are diagnosed with HIV. Sexual transmission of HCV is less likely, but several outbreaks of acute hepatitis C (ACH) have been described in HIV-positive MSM [16,17]. ACH is often asymptomatic or causes nonspecific symptoms, but suspicion of ACH may be raised because of elevated liver-enzymes when patients come for their routine check-ups. Between 2002 and 2010, we have observed 150 EuroSIDA patients who seroconverted from HCV antibody-negative to HCV antibody-positive, indicating the occurrence of acute infection. Two-thirds of these cases were among MSM while only 16 cases were among PWID. The incidence of ACH increased over time in all EuroSIDA regions and was, despite the low total number of cases, highest among PWID [18].

Persons who clear HCV-RNA either spontaneously or due to HCV treatment are at risk of becoming re-infected with HCV if they have continuous risk behavior. To investigate the occurrence of HCV re-infection in EuroSIDA patients, we measured HCV-RNA in the latest available follow-up plasma sample in all patients with prior spontaneous clearance included in an earlier study [14]. Thirty-five of 191 eligible patients (18%) had detectable HCV-RNA after a median 3.6 years of follow-up between the first and second HCV-RNA test. Thirty-three (94%) were PWID and two (6%) were MSM. Risk factors for having HCV-RNA recurrence were PWID, younger age and not receiving cART [11].

## Mortality-related findings

In the period from 2000 to 2013, around a quarter of all deaths among HCV treatment naïve co-infected patients could be attributed to their HCV infection. Other important causes of death among co-infected patients are AIDS (24%), cardiovascular disease (9%), infections (8%) and cancer (5%). The rate of liver-related death (LRD) peaked at age 35-45, and was two to four times lower in East and East Central compared with other EuroSIDA regions. Both lower CD4 cell count and elevated HIV-RNA were associated with increased risk of LRD. For example, the 5-year risk of LRD in patients with ≥F2 fibrosis (versus <F2) increased from 27-fold to 50-fold in patients with a CD4 cell count ≥200 cells/microliter and <200 cells/microliter, respectively [19].

A EuroSIDA study from 2005, which included both anti-HCV positive and HBsAg positive patients, found an average annual decrease in the rate of LRD of 7% from 1994 to 2004. The decrease could be explained by increases in the CD4 cell count after starting cART. However, in patients with similar CD4 cell counts the rate of LRD increased by 13% per year of additional exposure to cART [4]. Ongoing analyses have found a continuous decrease in LRD since 2004 in HCV treatment naïve patients. The reasons for this development are currently being explored.

In contrast to HBV infection, where there is evidence of an association between higher HBV-DNA levels and risk of liver-related death [20], studies investigating the association between HCV-RNA levels and risk of liver-related death have produced conflicting results. In general, those studies have been limited by small sample sizes and low numbers of clinical events [21,22]. A EuroSIDA study by Rockstroh et al found no effect of level of HCV-RNA or genotype on risk of liver-related death (N=86) among 2,709 HCV-RNA positive patients. However, patients who were HCV antibody-positive and HCV-RNA negative had a five-fold higher risk of liver-related death compared with HCV antibody-negative persons. This excess risk of LRD among patients with resolved HCV infection could partly be explained by a high prevalence of hepatitis B virus infection in HCV-RNA negative patients [13].

## Plasma hyaluronic acid: a strong predictor of liver-related clinical events and an important research tool in EuroSIDA

Historically, liver biopsy has been considered the gold standard for diagnosis and monitoring of the progression of fibrosis in patients with chronic viral hepatitis and other liver diseases. However, the drawbacks of liver biopsy are its invasiveness, imprecision and cost. Non-invasive low-cost methods to evaluate liver fibrosis are therefore needed.

**Figure 2:**
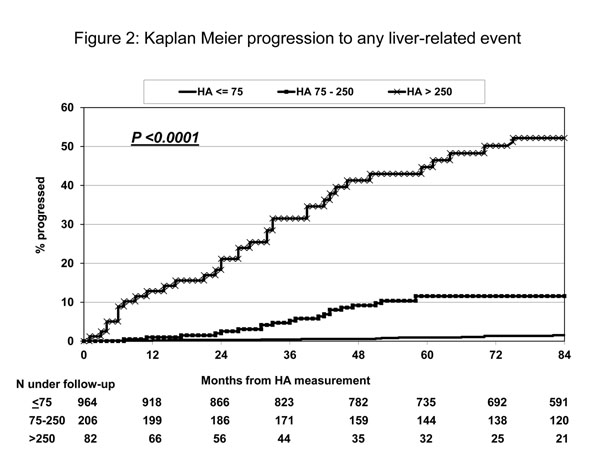
**Kaplan Meier progression to liver-related death or hepatic encephalopathy according to baseline plasma hyaluronic acid (HA) level** The figure shows the cumulative risk of liver-related death or development of hepatic encephalopathy for patients divided into three different groups according to the HA level (ng/ml) measured in the first available stored plasma sample from each patient. For patients with HA ≤75 ng/ml (the upper limit of normal) the 5-year risk was only 1%, while patients with HA>250 ng/ml the risk was nearly 45%. Among the included patients 67% were anti-HCV positive, the remaining patients had chronic hepatitis B.

EuroSIDA researchers assessed the reliability of plasma hyaluronic acid (HA) as a predictor of the risk of liver-related events (liver-related death and hepatic encephalopathy) in all patients who were positive for either HCV antibodies or hepatitis B surface antigen (HBsAg) and had stored plasma samples available [10]. Among 1252 included patients, 84 developed a liver-related event during a median of 8.2 years of follow-up. Patients with HA levels below 75 ng/ml (the normal range) in their first available plasma sample had a cumulative five-year risk of experiencing a liver-related event of only 1%, whereas the risk was 12% and 45% for patients with HA levels between 75-250 ng/ml and >250 ng/ml, respectively (Figure 2). Furthermore, patients who experienced a liver-related event and were tested for HA prior to their event had a median annual increase in HA of 108 ng/ml per year, whereas HA levels remained stable over time in a random control group of 172 patients from the same cohort, who did not develop a liver-related event.

Previous fibrosis marker research in hepatitis C has mainly been cross-sectional and has lacked relevant clinical end points. The results from EuroSIDA show that plasma HA may serve as a useful clinical tool for monitoring the progression of liver disease. Furthermore, HA has also proven to be an important research tool in EuroSIDA and other large studies, where a good estimate of liver disease severity is often lacking.

## Non-liver-related clinical events: is HCV the culprit or a marker of risk behavior?

HCV infection has been associated with an increased risk of chronic kidney disease (CKD) and other non-liver-related clinical events.[12] However, since most studies investigating these associations have based the hepatitis C diagnosis only on HCV antibody status, it is possible that the excess risk is due to other risk factors among HCV antibody-positive persons (e.g., injecting drug use) and not HCV *per se*. Since a large number of anti-HCV positive patients in EuroSIDA are well-characterized both in terms of HCV-RNA status and fibrosis, two studies have been conducted to disentangle the relative importance of these factors.

In one study the incidence of CKD was compared across three groups of HIV-positive patients: those who were HCV antibody negative, those who had resolved HCV infection (anti-HCV positive and HCV-RNA negative) and those who were anti-HCV positive/HCV-RNA positive. CKD was defined as a confirmed (> 3 months apart) estimated glomerular filtration rate of ≤60 ml/minute per 1.73 m^2^ (measured by the Cockcroft-Gault equation). Whereas there was no difference in incidence of CKD between the first two groups, patients with chronic HCV infection had a two-fold increased incidence of CKD (Incidence Rate Ratio 2.12; 95% CI 1.60 – 2.82; p<0.0001). The incidence of HCV did not vary by HCV genotype. Different HCV-related nephropathies have been described, but this study was not able to characterize the type of CKD [9].

Earlier EuroSIDA studies and other observational studies have found that anti-HCV positive persons more frequently discontinued antiretroviral drugs due to toxicity or physician choice compared with HCV negative patients [5,6,23]. Again, this could be due to HCV-related liver disease (since many antiretroviral drugs are metabolized in the liver) or due to other factors not directly related to HCV*.* A recent EuroSIDA study by Grint et al found that patients with chronic HCV infection had a 26% higher risk of antiretroviral drug discontinuation compared with patients with resolved HCV infection (IRR 1.26; 95% CI 1.06–1.50), but that the effect seems to be explained by more advanced liver fibrosis (as measured by elevated HA levels) in patient with chronic infection [3]. The effect of fibrosis on risk of drug discontinuation was mainly seen for the protease inhibitors (IRR 1.50; 95% CI 1.08–2.08; p=0.015) and the NRTIs (IRR 1.44; 95% CI 1.05–2.00; p=0.022), in particular the older NRTIs zidovudine and didanosine. Interestingly, among the different causes of toxicity, gastrointestinal toxicity was most common (35%) while only 7% was due to hepatotoxicity.

## Uptake of HCV treatment in EuroSIDA: are the right patients being targeted?

Until 2011, the standard of care for HIV/HCV co-infected patients in need of HCV treatment was weekly subcutaneous injections of pegylated interferon along with ribavirin taken orally twice per day for 48 weeks. This treatment eradicated the virus in about 30% to 70% of cases depending on the HCV genotype [24,25]. Due to the cost and the potential for adverse effects, most guidelines only recommended treatment in patients who had developed significant liver fibrosis and therefore were at risk of liver-related complications.

**Figure 3:**
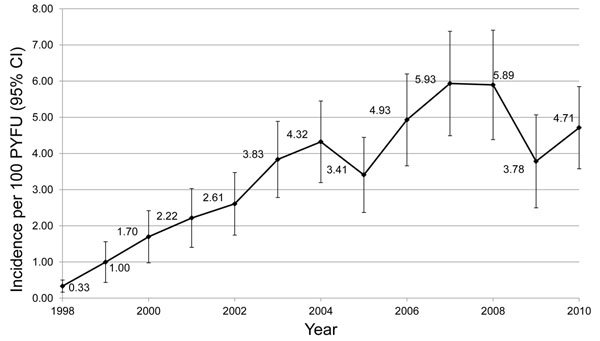
**Temporal change in Incidence of uptake of HCV treatment among EuroSIDA patients** In the period from 1998 -2007 the uptake of HCV treatment increased on average 27% per year (unadjusted incidence rate ratio: 1.27 (95% CI: 1.23 -1.31). From 2007 – 2010 the uptake of HCV decreased 12% per year (unadjusted IRR: 0.88 (95% CI: 0.79 -0.98).

In EuroSIDA a quarter of co-infected patients received HCV treatment between 1998 and 2010. On average, the incidence of HCV treatment increased 27% per year between 1998 and 2007 before falling on average 12% per year between 2007 and 2010 (figure 3). Patients residing in southern Europe have been more likely to receive HCV treatment compared with patients from other EuroSIDA regions. The decrease in recent years is probably explained by treatment saturation of the patients in urgent need of therapy, as well as by patients and physicians waiting for better treatment options [2,7]. Among the proportion of co-infected patients with information on fibrosis available, only one-third of treated patients had significant fibrosis (METAVIR F2 or greater), while 22% of untreated patients had significant fibrosis. Hence many patients with minimal short- to intermediate-term risk of HCV-related complications received a costly treatment associated with many adverse effects, while many patients at risk of complications of their HCV infection did not receive relevant treatment [2].

## Discussion

The EuroSIDA study follows one of the largest cohorts of HIV/HCV co-infected patients in the world, and is the only cohort to include patients from all geographical regions in Europe. The overall prevalence of HCV seropositivity among patients tested in EuroSIDA is 32.4% with the highest burden of co-infection seen in Eastern Europe where IDU is the most common mode of HIV transmission.

The large number of patients, detailed virological and clinical characterization and long-term follow-up have enabled EuroSIDA researchers to describe important regional differences in treatment and the long-term clinical outcome of HIV/HCV co-infected patients in Europe, but also to disentangle the relative importance of viral replication, hepatic fibrosis and other risk factors for some extra-hepatic conditions, such as chronic kidney disease, seen more commonly in this complex patient population.

Most co-infected patients in EuroSIDA are already HCV infected at the time of enrolment into EuroSIDA, but we have, in agreement with other cohort studies, documented a high risk of both primary HCV infection and HCV re-infection among PWID and MSM after enrolment in EuroSIDA. These findings underline importance of maintaining focus on preventive measures to reduce injecting drug use, sharing of contaminated needles and unprotected sex, but also that clinicians should maintain a high vigilance to identify patients with new HCV infection early and provide counseling to minimize the risk of onwards transmission and consider the need for HCV treatment.

In the period from 2000 to 2013, around a quarter of all deaths among HCV treatment naïve co-infected patients could be attributed to their HCV infection, and despite the ageing of the population and hence longer time for liver fibrosis to develop, HCV-related mortality has decreased in the period. Reasons for this development, which could be due to more patients with advanced liver fibrosis accessing HCV treatment, better control of the HIV infection or competing risks of death, especially in Eastern Europe (where the HCV epidemic is also more recent), are currently being explored.

Striking regional differences in causes of death were observed, with AIDS and non-liver-related death dominating in Eastern Europe whereas rates of liver-related death still remained low compared with other European regions. The same study confirmed that lower CD4 cell counts, HIV viral replication and HBV co-infection are all risk factors for liver-related death. These findings highlight the importance of HIV treatment as part of an integrated strategy to decrease the burden of not only HIV-related death, but also HCV-related death among HIV/HCV co-infected patients.

Until now, HCV treatment is likely to have improved the overall mortality of co-infected patients in EuroSIDA only minimally. We have shown that only a quarter of all co-infected patients in EuroSIDA have ever received interferon-based therapy, and among those who were treated only a third had evidence of significant liver fibrosis. On the other hand, 22% of all untreated patients have significant fibrosis. The reasons for the latter could be lack of access to or contraindications to therapy or fear of adverse effects of interferon and ribavirin.

With the recent approval of potent and well-tolerated oral direct acting antivirals (DAAs) against HCV and the prospect of interferon-free treatment for all HCV genotypes, new HCV therapy could potentially eradicate the virus in nearly all treated patients and sharply reduce HCV related mortality [26]. However, given the exorbitant prices of DAAs, it is already clear now that unless their cost is reduced substantially, DAA-based therapy will be inaccessible for many HCV infected patients in many parts of Europe.

Monitoring of these important regional differences in uptake of DAA-based therapy across Europe, and to investigate the short- and long-term efficacy and adverse effects of these new drugs in a “real-life” co-infected population as compared with the generally healthier patients selected for clinical trials, will be an important future research priority for the EuroSIDA study.

## Ethics and consent

Before any study related activities are performed Local Ethical Committee approval of the study and procedure for obtaining informed consent from participants is obtained according to local and/or national regulations in all countries participating in the study as well as other national regulatory approvals as applicable. The senior investigator at each clinical site is responsible for obtaining and maintaining this/these approval(s) at all times during the conduct of the study.

## Funding

Primary support for EuroSIDA is provided by the European Commission BIOMED 1 (CT94–1637), BIOMED 2 (CT97–2713), the 5th Framework (QLK2–2000-00773) and the 6th Framework (LSHPCT- 2006-018632), and the 7th Framework (FP7/2007- 2013, EuroCoord n*8* 260694) programs. Current support also includes unrestricted grants by Gilead, Pfizer, BMS, Merck and Co. The participation of centers from Switzerland was supported by The Swiss National Science Foundation (Grant 108787).

## Abbreviations

PYFU: person-years of follow-up; CI: confidence interval

## Competing interests

The authors declare that they have no competing interests.

## Declarations

This article has been published as part of *BMC Infectious Diseases* Volume 14 Supplement 6, 2014: Viral Hepatitis in Europe. The full contents of the supplement are available online at http://www.biomedcentral.com/bmcinfectdis/supplements/14/S6. The publication charges for this supplement were funded by AbbVie as an unrestricted grant to Rigshospitalet, the University of Copenhagen. AbbVie further funded the printing of the supplement with additional financial support from the Drug Prevention and Information Programme (DPIP) of the European Union.
